# Antimicrobial use among paediatric inpatients at hospital sites within the Canadian Nosocomial Infection Surveillance Program, 2017/2018

**DOI:** 10.1186/s13756-023-01219-x

**Published:** 2023-04-18

**Authors:** Wallis Rudnick, John Conly, Daniel J. G. Thirion, Kelly Choi, Linda Pelude, Joelle Cayen, John Bautista, Lizanne Beique, Jeannette L. Comeau, Bruce Dalton, Johan Delport, Rita Dhami, Joanne Embree, Yannick Émond, Gerald Evans, Charles Frenette, Susan Fryters, Jennifer Happe, Kevin Katz, Pamela Kibsey, Joanne M. Langley, Bonita E. Lee, Marie-Astrid Lefebvre, Jerome A. Leis, Allison McGeer, Susan McKenna, Heather L. Neville, Kathryn Slayter, Kathryn N. Suh, Alena Tse-Chang, Karl Weiss, Michelle Science, the Canadian Nosocomial Infection Surveillance Program

**Affiliations:** 1 Public Health Agency of Canada, 130 Colonnade Rd, Ottawa, ON K2E 7L9 Canada; 2 University of Calgary, 3330 Hospital Dr NW, Calgary, AB T2N 4N1 Canada; 3 Foothills Medical Centre, Alberta Health Services, 3330 Hospital Dr. NW, Calgary, AB T2N 2T9 Canada; 4 Université de Montréal, 2900 Boulevard Edouard-Montpetit, Montréal, QC H3T 1J4 Canada; 5 McGill University Health Centre, 1001 Boulevard Décarie, Montréal, QC H4A 3J1 Canada; 6 Central Newfoundland Regional Health Centre, 50 Union, Grand Falls-Windsor, NL A2A 2E1 Canada; 7 IWK Health Centre, 5980 University Ave, Halifax, NS B3K 6R8 Canada; 8 Dalhousie University, 6299 South St, Halifax, NS B3H 4R2 Canada; 9 Alberta Health Services, 1620 29 St NW, Calgary, AB T2N 4L7 Canada; 10 London Health Sciences Centre, 800 Commissioners Rd E, London, ON N6A 5W9 Canada; 11 University of Waterloo, 200 University Ave W, Waterloo, ON N2L 3G1 Canada; 12 University of Western Ontario, 1151 Richmond St, London, ON N6A 3K7 Canada; 13 University of Manitoba, Winnipeg, MB R3T 2N2 Canada; 14 Shared Health Manitoba, Winnipeg, MB R3T 2N2 Canada; 15 Children’s Hospital Winnipeg, 840 Sherbrook St, Winnipeg, MB R3E 0Z3 Canada; 16 Hôpital Maisonneuve-Rosemont, 5415 Boulevard de l’Assomption, Montréal, QC H1T 2M4 Canada; 17 Kingston Health Sciences Centre, 76 Stuart St, Kingston, ON K7L 2V7 Canada; 18 Alberta Health Services, 10240 Kingsway Avenue, Edmonton, AB T5H 3V9 Canada; 19 Infection Prevention and Control Canada, Red Deer, AB T4N 6R2 Canada; 20 North York General Hospital, 4001 Leslie St, North York, ON M2K 1E1 Canada; 21 Royal Jubilee Hospital, 1952 Bay St, Victoria, BC V8R 1J8 Canada; 22 Stollery Children’s Hospital, Edmonton, AB T6G 2B7 Canada; 23 University of Alberta, Edmonton, AB T6G 2R7 Canada; 24 Department of Medicine, University of Toronto, 1 King’s College Cir, Toronto, ON M5S 1A8 Canada; 25 Sunnybrook Health Sciences Centre, 2075 Bayview Ave, Toronto, ON M4N 3M5 Canada; 26 Sinai Health System, 600 University Ave, Toronto, ON M5G 1X5 Canada; 27 University of Toronto, 27 King’s College Cir, Toronto, ON M5S 1A1 Canada; 28 Dalla Lana School of Public Health, University of Toronto, 155 College St, Toronto, ON M5T 3M7 Canada; 29 Nova Scotia Health, 1276 South Park St, Halifax, NS B3H 2Y9 Canada; 30 The Ottawa Hospital, 501 Smyth Rd, Ottawa, ON K1H 8L6 Canada; 31 SMBD-Jewish General Hospital, 3755 Chemin de la Côte-Sainte-Catherine, Montréal, QC H3T 1E2 Canada; 32 SickKids, 555 University Ave, Toronto, ON M5G 1X8 Canada

**Keywords:** Antimicrobial use, Hospital, Paediatric, Surveillance

## Abstract

**Background:**

Antimicrobial resistance threatens the ability to successfully prevent and treat infections. While hospital benchmarks regarding antimicrobial use (AMU) have been well documented among adult populations, there is less information from among paediatric inpatients. This study presents benchmark rates of antimicrobial use (AMU) for paediatric inpatients in nine Canadian acute-care hospitals.

**Methods:**

Acute-care hospitals participating in the Canadian Nosocomial Infection Surveillance Program submitted annual AMU data from paediatric inpatients from 2017 and 2018. All systemic antimicrobials were included. Data were available for neonatal intensive care units (NICUs), pediatric ICUs (PICUs), and non-ICU wards. Data were analyzed using days of therapy (DOT) per 1000 patient days (DOT/1000pd).

**Results:**

Nine hospitals provided paediatric AMU data. Data from seven NICU and PICU wards were included. Overall AMU was 481 (95% CI 409–554) DOT/1000pd. There was high variability in AMU between hospitals. AMU was higher on PICU wards (784 DOT/1000pd) than on non-ICU (494 DOT/1000pd) or NICU wards (333 DOT/1000pd). On non-ICU wards, the antimicrobials with the highest use were cefazolin (66 DOT/1000pd), ceftriaxone (59 DOT/1000pd) and piperacillin-tazobactam (48 DOT/1000pd). On PICU wards, the antimicrobials with the highest use were ceftriaxone (115 DOT/1000pd), piperacillin-tazobactam (115 DOT/1000pd), and cefazolin (111 DOT/1000pd). On NICU wards, the antimicrobials with the highest use were ampicillin (102 DOT/1000pd), gentamicin/tobramycin (78 DOT/1000pd), and cefotaxime (38 DOT/1000pd).

**Conclusions:**

This study represents the largest collection of antimicrobial use data among hospitalized paediatric inpatients in Canada to date. In 2017/2018, overall AMU was 481 DOT/1000pd. National surveillance of AMU among paediatric inpatients is necessary for establishing benchmarks and informing antimicrobial stewardship efforts.

## Background

The advent of antibiotics has saved many lives and has created the conditions for much of modern medicine [1]. However, overuse of antibiotics has led to the emergence of antimicrobial resistant organisms [2], currently threatening our ability to prevent and treat infections. Although hospital benchmarks for antimicrobial use (AMU) have been well documented among adult populations [3], less attention has been paid to paediatric inpatients. On an individual patient level, paediatric antibiotic exposure may lead to negative repercussions for child and adult health [4–8].

Antibiotic use is very common among hospitalized children [9]. In studies from North America and Europe, 29–61% of hospitalized paediatric patients receive antibiotics [10–13]. Data from our network of Canadian acute care hospitals indicate that 56% of hospitalized children aged 1–17 years received antibiotics in a 2017 point prevalence study [14].

Among hospitalized paediatric patients, it is estimated that potentially 9–43% of prescriptions are unnecessary or inappropriate [15–18]. Misuse of antibiotics among neonatal and paediatric wards has been associated with adverse patient outcomes including increased risk of infection with resistant organisms [19–27].

Antimicrobial stewardship programs aim to find a balance between the “potent ability of antibiotics for individual patients and their potentially hazardous effects” [28]. Paediatric stewardship programs optimize how and when antimicrobials are used and have been shown to reduce inappropriate prescriptions [29] and to reduce antibiotic consumption [30–33]. Paediatric antimicrobial stewardship programs can improve patient outcomes and reduce costs [34, 35]. In 2013, implementing an antimicrobial stewardship program became a requirement of accreditation for all Canadian acute-care hospitals [36]; in 2018, 93% of surveyed academic paediatric hospitals in Canada had a formal antimicrobial stewardship program [37].

Antimicrobial use (AMU) surveillance can identify opportunities for interventions, enable evaluation of antimicrobial stewardship programs and help garner political will for successful stewardship campaigns [38].

There are published AMU data from a paediatric hospital [39] and from five NICU wards [40] in Alberta, otherwise data on paediatric AMU in Canadian hospitals are limited. National point prevalence studies have provided estimates of the prevalence of paediatric patients receiving therapy from a snapshot in time [14] as well as estimates of days of therapy [13]. To address these data gaps, the Canadian Nosocomial Infection Surveillance Program (CNISP) developed a paediatric AMU surveillance program for acute-care secondary and tertiary hospitals across Canada with the following three objectives: (1) estimate national paediatric AMU in secondary and tertiary care hospitals; (2) provide AMU benchmarks for paediatric wards; and (3) estimate AMU by ward-type.

## Methods

### Setting and participating sites

CNISP is a collaboration between the Canadian Hospital Epidemiology Committee, a subcommittee of the Association of Medical Microbiologists and Infectious Disease, and the Public Health Agency of Canada. As of January 2022, 89 sentinel hospitals, from across 10 provinces and one territory participate in the CNISP network. Forty hospitals serve paediatric inpatients; nine are standalone paediatric hospitals.

CNISP established a working group for antimicrobial use in 2007/08. Paediatric AMU surveillance started as a pilot study among a few hospitals before transitioning to routine surveillance. The results of this current study represent the nine hospitals that participated in CNISP paediatric AMU surveillance in 2017 and/or 2018.

### Data variables and collection

#### Paediatric inpatients

Paediatric patients were defined as those < 18 years of age or those patients on wards where the majority of patients are < 18 years of age. Surveillance included all acute care inpatient units (including intensive care units) and admissions in emergency departments. Non-admitted patients in emergency departments were excluded. Participating sites provided corresponding paediatric inpatient-day denominators by ward. Estimates of national inpatient days by year and age group were obtained from the Canadian Institute for Health Information [41].

#### Antimicrobial use

Participating hospitals provided total paediatric inpatient AMU separated by type of antimicrobial and ward category (NICU, PICU and non-ICU wards). Hospitals were asked to submit either dispensed or administered antimicrobials and to separate their data by administration route (parenteral and oral) if possible. All systemic antibacterial use was included in the surveillance using Anatomical Therapeutic Chemical (ATC) codes: J01s, P01AB01 (metronidazole oral) and A07AA09 (oral vancomycin) [42]. Quantity of antimicrobials were submitted as days of therapy (DOT), defined as the number of days that a patient receives an antimicrobial agent regardless of dose. The DOT for a given patient on multiple antibiotics is the sum of DOTs for each antibiotic that the patient is receiving.

#### Data analysis

Participating hospitals submitted annual data files. The WHO ATC/DDD Index [42] was adapted in order to group antimicrobials by drug class. AMU data were used to rank the most frequently prescribed antimicrobial agents by drug class and by ward type. Relative differences were calculated by taking the difference between two rates and dividing the difference by the smaller rate. National rates of AMU were calculated and standardized per 1000 inpatient days (pd): rates were calculated as (total DOTs / total pd) * 1000. Bootstrapped standard errors with 10,000 replications were used to calculate 95% confidence intervals (95% CI). All analyses were done using SAS (version 9.4) software.

## Results

### Participating sites

Nine CNISP hospitals provided paediatric AMU data. Eight hospitals provided data for both 2017 and 2018 calendar years; one hospital provided data only for 2018. Total inpatient days included in surveillance (507 583 patient days) represented about a quarter of paediatric inpatient days in Canada in 2017/18. Three participating hospitals were in western Canada, four in central Canada (Ontario/Quebec), and two in eastern Canada. Five of the hospitals were paediatric acute care centres with ≤ 200 beds and four hospitals were mixed adult/paediatric hospitals with 201–500 beds. Seven PICUs and seven NICUs were included in surveillance. PICUs and NICUs represented 9% and 23% of included patient days, respectively. Participating site characteristics are summarized in Table 1.

**Table 1:** Characteristics of hospitals and intensive care units (ICUs) participating in surveillance of paediatric antimicrobial use, 2017–2018

Characteristic	Hospitals	non-ICU wards	Neonatal ICUs	Paediatric ICUs*
Number of hospitals submitting data	9	9	7	7
Hospital sites				
Paediatric hospitals	5	5	5	5
Mixed (adult/paediatric) hospitals	4	4	2	2
Paediatric Inpatient Days	507,583	344,073	118,949	44,561
Total days of therapy	244,373	169,830	39,592	34,951
Regions				
West	3	3	2	2
Central	4	4	4	3
East	2	2	1	2
Hospital bed size				
201–500 beds	4	4	2	2
≤ 200 beds	5	5	5	5
Hospital type				
Teaching	9	9	7	7
Community	0	0	0	0

*Includes one paediatric cardiovascular ICU

### Antimicrobial use

**Fig. 1:**
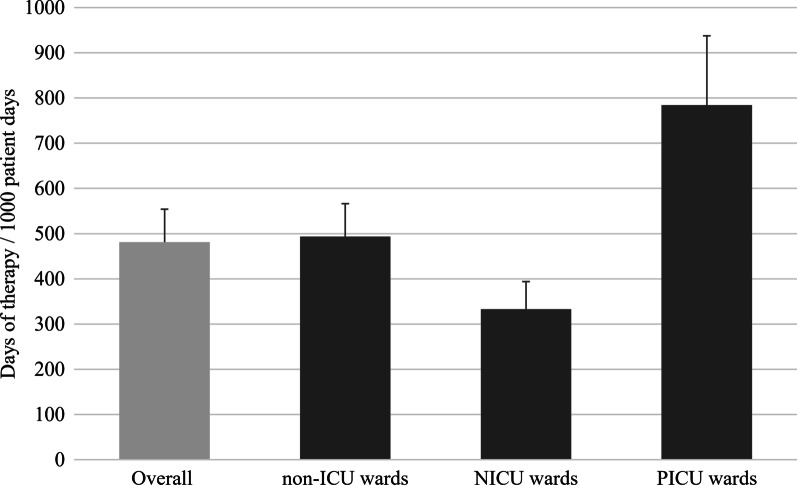
Rate of antimicrobial use among paediatric inpatients overall and by ward type with bootstrapped 95% confidence intervals, 2017–2018

From January 2017 to December 2018, total AMU was 481 (95% CI 409–554) DOT/1000 patient days (/1000pd). AMU varied substantially between hospitals; the interquartile range (IQR) for total AMU spanned 217 DOT/1000pd: 352–569 DOT/1000pd and there was 17-fold variability between hospitals’ rates of overall AMU (Fig. 1). Among the eight hospitals that provided two years of data, overall AMU rates differed on average by 10% between the two years (range < 1% to 24%); three hospitals had higher rates in 2018 than 2017 and five hospital had lower rates in 2018. Overall, AMU declined by 9% between 2017 and 2018, however, this was not statistically significant (difference: − 44 DOT/1000pd; 95% CI: − 101–13 DOT/1000pd).

**Fig. 2:**
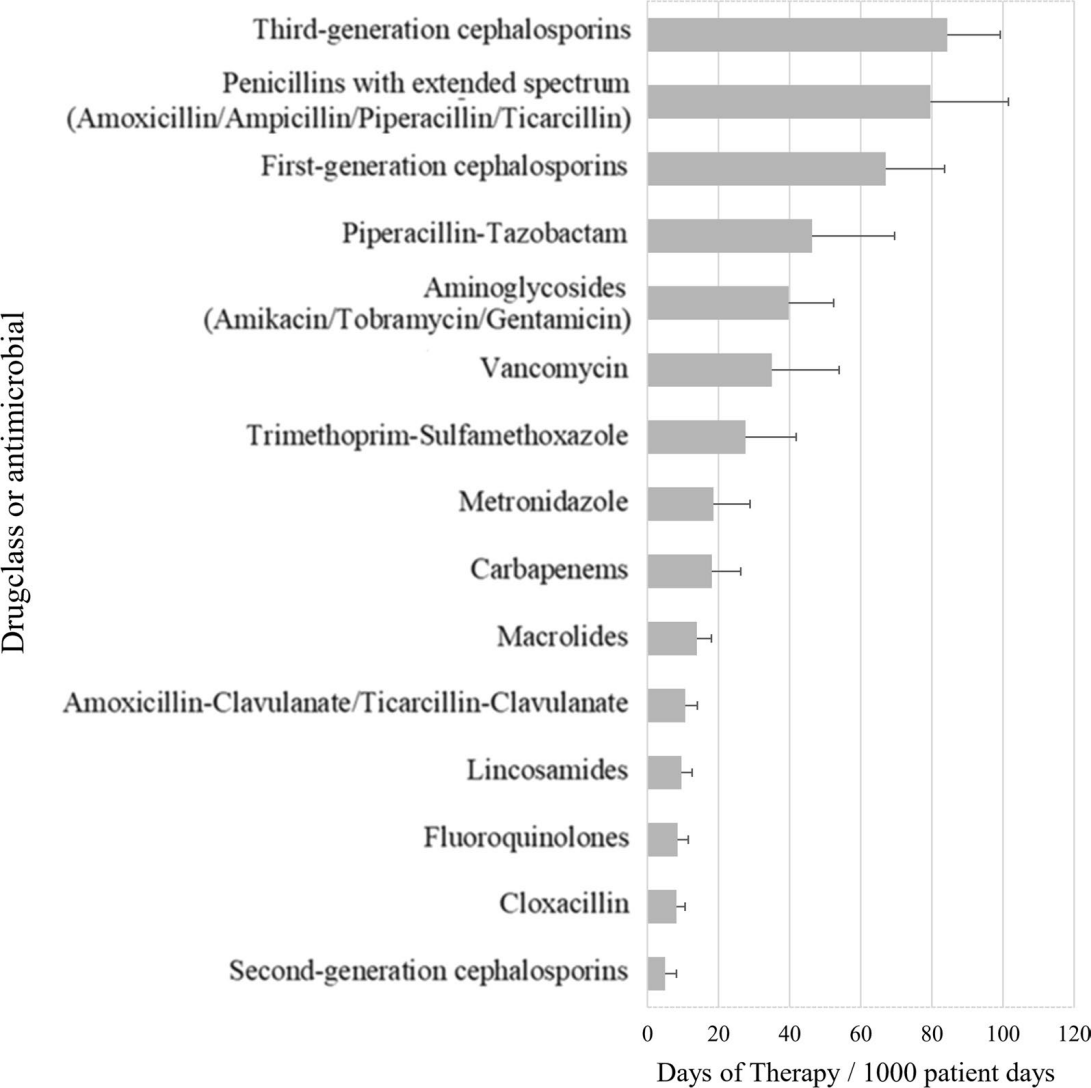
Rate of antimicrobial use among paediatric inpatients by drug class with bootstrapped 95% confidence intervals, 2017–2018. Presented antimicrobials represent 98% of reported antimicrobials used at participating hospitals

The classes of antimicrobials with the highest use (Fig. 2) were the third-generation cephalosporins (84 DOT/1000pd), penicillins with extended spectrum (80 DOT/1000pd; including amoxicillin, ampicillin, piperacillin and ticarcillin), first-generation cephalosporins (67 DOT/1000pd), piperacillin-tazobactam (46 DOT/1000pd), and aminoglycosides (40 DOT/1000pd including amikacin, tobramycin, and gentamicin).

**Fig. 3:**
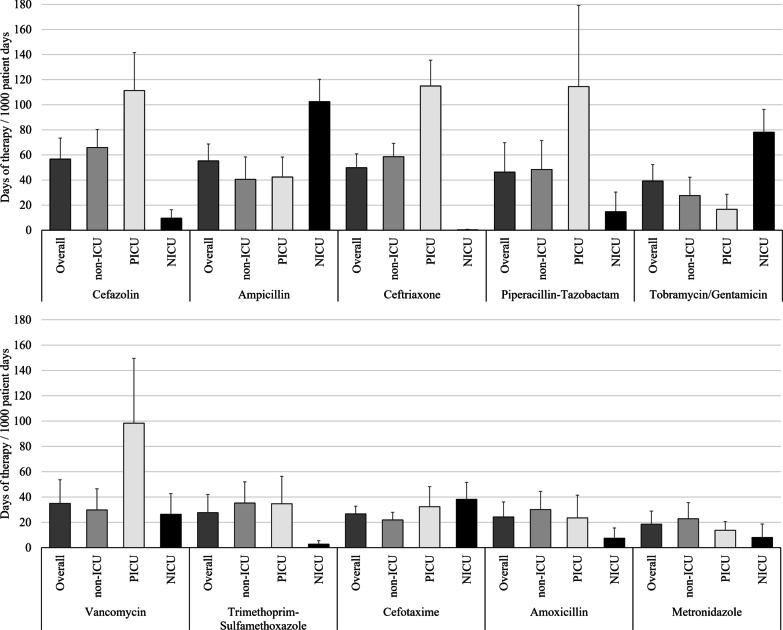
Rate of antimicrobial use among paediatric inpatients for the 10 most used antimicrobials with bootstrapped 95% confidence intervals, 2017–2018. Presented antimicrobials represent 80% of reported antimicrobials used at the participating hospitals

Including all clinical units from the participating sites, the most frequently used antimicrobials (Fig. 3) were cefazolin (57 DOT/1000pd), ampicillin (55 DOT/1000pd), ceftriaxone (50 DOT/1000pd), piperacillin-tazobactam (46 DOT/1000pd), tobramycin/gentamicin (39 DOT/1000pd), vancomycin (oral and parenteral combined, 35 DOT/1000pd), trimethoprim-sulfamethoxazole (28 DOT/1000pd), cefotaxime (27 DOT/1000pd), amoxicillin (24 DOT/1000pd), and metronidazole (19 DOT/1000pd). These 10 antimicrobials represented 79% (379/481 DOT) of total AMU. At the three hospitals where oral vancomycin use could be separated from parenteral use, 8% of vancomycin use was oral (3 DOT/1000pd).

Although AMU among PICUs represented only a small proportion of the total AMU (14% of overall DOTs), the rate of AMU was more than 50% higher among PICUs (784 DOT/1000pd) than among non-ICU wards (494 DOT/1000pd, *p*-value < 0.01). Among the seven PICUs included in surveillance, the interquartile range (IQR) for total AMU spanned from 502 to 900 DOT/1000pd. The ten most frequently used antimicrobials among PICUs were ceftriaxone (115 DOT/1000pd), piperacillin-tazobactam (115 DOT/1000pd), cefazolin (111 DOT/1000pd), vancomycin (98 DOT/1000pd oral and parenteral combined), meropenem (44 DOT/1000pd), ampicillin (42 DOT/1000pd), azithromycin (41 DOT/1000pd), trimethoprim-sulfamethoxazole (35 DOT/1000pd), cefotaxime (32 DOT/1000pd) and gentamicin/tobramycin (25 DOT/1000pd). These ten antimicrobials represented 84% of total AMU among PICUs.

Among the 20 most frequently used antimicrobials, antimicrobials with the largest relative differences between rates of use among PICUs and among non-ICU wards were vancomycin, meropenem and azithromycin; for these antimicrobials, use was 2–3 × higher on PICUs compared to non-ICUs. Although the rate of vancomycin use was much higher on PICU wards from seven hospitals (98 DOT/1000pd) compared to non-ICU wards from nine hospitals (30 DOT/1000pd), among the three hospitals with available data, the rate of oral vancomycin use was higher among non-ICU wards (5 DOT/1000pd) than among PICU wards (3 DOT/1000pd). Only cephalexin, metronidazole, ceftazidime and amoxicillin were used substantively more frequently among non-ICU wards compared with PICU wards; cephalexin use was 65% higher on non-ICU wards, metronidazole use was 40% higher, ceftazidime use was 28% higher and amoxicillin use was 22% higher.

The rate of total AMU among the seven NICUs (333 DOT/1000pd) was lower than on non-ICU wards (494 DOT/1000pd). Among the seven NICUs included in surveillance, the interquartile range (IQR) for total AMU spanned from 296 to 437 DOT/1000pd. The five antimicrobials used most often on NICUs were ampicillin (103 DOT/1000pd), gentamicin/tobramycin (78 DOT/1000pd), cefotaxime (38 DOT/1000pd), vancomycin (IV, 26 DOT/1000pd), and meropenem (16 DOT/1000pd). These five antimicrobials represented 78% of AMU among NICUs.

## Discussion

To date, these surveillance results represent the largest collection of dispensed or administered antibiotic use data from hospitalized paediatric patients in Canada. From January 2017 to December 2018, among hospitalized paediatric patients, the rate of overall AMU was 481 DOT/1000pd with substantial variation between hospitals and between ward types.

AMU data from hospitalized paediatric populations are limited and differences in methods (eg. metrics to express AMU), services, and patient populations make national and international comparisons difficult. However, there are studies that report paediatric AMU rates similar to those in this study (IQR: 352–569 DOT/1000pd). A study of 20 hospitals in the United States reported an overall annual paediatric AMU rate of 540 DOT/1000pd in 2007 [43]. A four-hospital point prevalence study in Italy estimated an overall paediatric AMU rate of 305 DOT/1000pd in 2016 [44].

There are also Canadian and international studies that report higher rates of paediatric AMU than those found in our study. Our AMU rate among non-ICU wards (494 DOT/1000pd) is 55% of the median-adjusted AMU rate found among non-ICU wards from 41 hospitals in the United States (893 DOT/1000pd from billing data) [45]. A Canadian study conducted at one of the hospitals included in this study using a similar methodology found an AMU rate of 757 DOT/1000pd in 2013/14 [39]. Differences in case mixes and included time periods may explain or partially explain the differences in rates; notably, the Canadian study found that rates of AMU were decreasing at their centre [39]. Among our nine hospitals, there was high variability in overall AMU rates with a 17-fold variability between hospitals and an interquartile range spanning 217 DOT/1000pd. The high variation between AMU rates at paediatric hospitals is not surprising given that paediatric AMU rates within the same jurisdiction have been found to vary widely [44, 46]. The variation observed in our study is likely at least partially attributable to differences in hospital services, clinical specialties and the presence of ICUs. Further study is needed to identify the reasons for this variability and how to optimize interventions in light of this variation.

In our study, the rate of AMU among PICU wards was about 1.5 times as high as the rate of AMU among non-ICU wards. Higher rates of AMU among PICU wards are expected due to the higher prevalence of infection among critically ill patients. Perioperative antibiotic prophylaxis, suspected ventilator-associated pneumonia and sepsis are drivers of AMU on PICU wards [47–49]. In addition, guidelines for antimicrobial use often involve recommendations for empiric use of more than one antimicrobial agent among PICU patients [50, 51]. Although our absolute rates of AMU were lower, a study from a hospital in Oregon reported about a twofold difference in AMU on a PICU ward compared to their non-ICU wards [32]. Some studies have found smaller differences in rates between PICU and non-ICU wards [45, 52] likely resulting in part from differences in services and clinical specialties at these institutions. Estimates of inappropriate antimicrobial use on PICUs vary widely ranging from 17 to 62% [47, 53]. It is notable that, despite the high rates of AMU on PICU wards, interventions in the PICU will impact only a small portion of total antibiotic use; PICUs represented 14% of total DOTs in our study.

Our rate of AMU among PICU wards (784 DOT/1000pd) was lower than most rates reported by others possibly due to the state of stewardship programs at these centres. A large study of billing data from 41 PICUs in the United States reported a median-adjusted rate of 1043/1000pd in 2010–2014 [45]. Studies from Saudi Arabia in the mid-2010s found AMU rates among PICUs between 697 and 849 DOT/1000pd [54, 55]. A German intervention study found an AMU rate of 1226 DOT/1000pd [49]. A 2015 study of AMU among a PICU in South Africa reported a rate of 1336 DOT/1000pd [52]. A study of German and Brazilian PICUs found rates of 888 and 1441 DOT/1000pd, respectively; patients with < 24 h of AMU were excluded in this study [56].

Glycopeptide use among PICUs (98 DOT/1000pd) was more than three-times higher than glycopeptide use among non-ICU wards (30 DOT/1000pd); this is likely due in part to more frequent use of central lines and coverage for coagulase negative staphylococci on PICUs. Glycopeptide use among PICUs in this study was similar to use on a German PICU (90 DOT/1000pd) [56], but lower than Brazilian, Saudi Arabian and South African PICUs (151 to 263 DOT/1000pd) [52, 54–56]. Differences in glycopeptide use may be partially due to differences in rates of methicillin-resistant *Staphylococcus aureus* across jurisdictions [56]. Vancomycin has also been found to represent a high percentage of inappropriate use in some jurisdictions [55, 57–59].

Our rate of overall AMU among NICUs (333 DOT/1000pd) is similar to some reports from Canada and the United States. Among five NICUs in Alberta, Canada, rates of AMU ranged from 155 to 624 DOT/1000pd in 2011–2014 [40]. In the United States, Cantey et al. found a decline from 343 DOT/1000pd in 2012 to 252 DOT/1000pd in 2014 after implementing a stewardship program [60]. Our rate was similar to that reported on a Saudi Arabian NICU in 2012–2015 (325 DOT/1000pd) [54] and was slightly lower than rates reported on two German NICUs (373–486 DOT/1000pd) in 2018 [56]. Much higher rates of AMU among NICU wards have been reported from other jurisdictions. Surveillance of a Brazilian NICU and five Russian NICUs found overall rates of AMU to be 1336 and 1423 DOT/1000pd, respectively [56, 61]. These differences may partially be due to differences in levels of NICUs; higher levels of NICU wards that provide more specialized care have been found to have higher rates of AMU than lower levels reflecting the underlying conditions (e.g. higher rates of surgical complications), severity of illness and risk of infection in more premature neonates, especially those with very low birth weight [40].

We acknowledge the limitations of our work including the risk of selection bias due to hospitals voluntarily opting to participate. The majority of the hospitals included had well-developed antimicrobial stewardship programs, which may not reflect all paediatric hospitals in Canada. Data were collected only from teaching hospitals and were not collected from every province so are not representative of all Canadian hospitals. We did not identify which hospitals or wards had patient groups with higher expected levels of AMU. Our surveillance system does not capture data on indication for use or appropriateness of use. There are also shortcomings to using DOTs to measure aggregate antibiotic use [62]. Interpretation of DOT data can be challenging given that it is not possible to separate monotherapy from combination therapy. The use of dispensed data may not represent what antibiotics were administered to the patients [63].

## Conclusions

Our study describes Canadian paediatric AMU data from nine hospitals and represents the largest collection of dispensed/administered antibiotic use data from paediatric inpatients in Canada to date. In 2017/2018, overall AMU was 481 DOT/1000pd. There is need for high-quality, hospital-based AMU surveillance to support antimicrobial stewardship efforts.

## Data Availability

The aggregate national-level datasets used and/or analysed during the current study are available from the corresponding author on reasonable request. The hospital-level datasets generated and/or analysed during the current study are not publicly available due to the binding data sharing agreements with the hospitals involved in the surveillance program.

## Abbreviations

AMU: Antimicrobial use
ATC: Anatomical therapeutic chemical
CI: Confidence interval
CNISP: Canadian nosocomial infection surveillance program
DOT: Days of therapy
DDD: Defined daily dose
ICU: Intensive care unit
NICU: Neonatal intensive care unit
PICU: Paediatric intensive care unit
PD: Patient days

## References

[1] Aminov RI (2010). A brief history of the antibiotic era: Lessons learned and challenges for the future. Front Microbiol.

[2] Cantón R, Morosini MI (2011). Emergence and spread of antibiotic resistance following exposure to antibiotics. FEMS Microbiol Rev.

[3] Rudnick W (2020). Antimicrobial use among adult inpatients at hospital sites within the Canadian Nosocomial Infection Surveillance Program: 2009 to 2016. Antimicrob Resist Infect Control.

[4] Rasmussen Msc SH (2018). Antibiotic exposure in early life and childhood overweight and obesity: a systematic review and meta-analysis. Diabetes Obes Metab.

[5] Marra F (2006). Does antibiotic exposure during infancy lead to development of asthma?* A systematic review and metaanalysis. Chest.

[6] Ni J (2019). Early antibiotic exposure and development of asthma and allergic rhinitis in childhood. BMC Pediatr.

[7] Yamamoto-Hanada K, Yang L, Narita M, Saito H, Ohya Y (2017). Influence of antibiotic use in early childhood on asthma and allergic diseases at age 5. Ann Allergy Asthma Immunol.

[8] Sarkar A (2021). The association between early-life gut microbiota and long-term health and diseases. J Clin Med.

[9] Gerber JS, Jackson MA, Tamma PD, Zaoutis TE, Committee on infectious diseases - pediatric infectious diseases society. Antibiotic Stewardship in Pediatrics. Pediatrics 2021;147.

[10] Versporten A (2016). The Worldwide Antibiotic Resistance and Prescribing in European Children (ARPEC) point prevalence survey: developing hospital-quality indicators of antibiotic prescribing for children. J Antimicrob Chemother.

[11] Potocki M, Goette J, Szucs TD, Nadal D (2003). Infection prospective survey of antibiotic utilization in pediatric hospitalized patients to identify targets for improvement of prescription. Infection.

[12] Ang L, Laskar R, Gray JW (2008). A point prevalence study of infection and antimicrobial use at a UK children’s hospital. J Hosp Infect.

[13] Blackburn J, Barrowman N, Bowes J, Tsampalieros A, Le Saux N (2021). Establishing benchmarks for antimicrobial use in Canadian children’s hospitals: results from 2 national point prevalence surveys. Pediatr Infect Dis J.

[14] Liang JJ (2022). Antimicrobial use in Canadian acute-care hospitals: Findings from three national point-prevalence surveys between 2002 and 2017. Infect Control Hosp Epidemiol.

[15] Tribble AC (2020). Appropriateness of antibiotic prescribing in United States children’s hospitals: a national point prevalence survey. Clin Infect Dis.

[16] McMullan BJ (2020). Antibiotic appropriateness and guideline adherence in hospitalized children: results of a nationwide study. J Antimicrob Chemother.

[17] Arcavi L (2010). Appropriate antibiotic prescribing pattern in hospitalized children. Curr Drug Saf.

[18] Goycochea-Valdivia WA (2017). Identifying priorities to improve paediatric in-hospital antimicrobial use by cross-sectional evaluation of prevalence and appropriateness of prescription. Enfermedades Infecc y Microbiol Clin (English ed).

[19] Michael Cotten C (2009). Prolonged duration of initial empirical antibiotic treatment is associated with increased rates of necrotizing enterocolitis and death for extremely low birth weight infants. Pediatrics.

[20] Alexander VN, Northrup V, Bizzarro MJ (2011). Antibiotic exposure in the newborn intensive care unit and the risk of necrotizing enterocolitis. J Pediatr.

[21] Cantey JB, Pyle AK, Wozniak PS, Hynan LS, Sánchez PJ (2018). Early antibiotic exposure and adverse outcomes in preterm, very low birth weight infants. J Pediatr.

[22] Kuppala VS, Meinzen-Derr J, Morrow AL, Schibler KR (2011). Prolonged initial empirical antibiotic treatment is associated with adverse outcomes in premature infants. J Pediatr.

[23] Novitsky A (2015). Prolonged early antibiotic use and bronchopulmonary dysplasia in very low birth weight infants. Am J Perinatol.

[24] Ting JY et al. Duration of initial empirical antibiotic therapy and outcomes in very low birth weight infants. Pediatrics 2019;143. 10.1542/peds.2018-2286 30819968

[25] Ting JY (2016). Association between antibiotic use and neonatal mortality and morbidities in very low-birth-weight infants without culture-proven sepsis or necrotizing enterocolitis. JAMA Pediatr.

[26] Ting JY (2018). Association of antibiotic utilization and neurodevelopmental outcomes among extremely low gestational age neonates without proven sepsis or necrotizing enterocolitis. Am J Perinatol.

[27] Cantey JB (2017). Antibiotic exposure and risk for death or bronchopulmonary dysplasia in very low birth weight infants. J Pediatr.

[28] Hulscher ME, Grol RP, van der Meer JW (2010). Antibiotic prescribing in hospitals: a social and behavioural scientific approach. Lancet Infect Dis.

[29] Donà D (2019). Implementation and impact of pediatric antimicrobial stewardship programs: a systematic scoping review. Antimicrob Resist Infect Control.

[30] Kreitmeyr K (2017). Pediatric antibiotic stewardship: successful interventions to reduce broad-spectrum antibiotic use on general pediatric wards. Infection.

[31] Simó S (2020). Effects of a paediatric antimicrobial stewardship program on antimicrobial use and quality of prescriptions in patients with appendix-related intraabdominal infections. Antibiotics.

[32] Turner RB, Valcarlos E, Loeffler AM, Gilbert M, Chan D (2017). Impact of an antimicrobial stewardship program on antibiotic use at a nonfreestanding children’s hospital. J Pediatric Infect Dis Soc.

[33] AraujodaSilva AR (2018). Role of antimicrobial stewardship programmes in children: a systematic review. J Hospital Infect.

[34] Dellit TH (2007). Infectious diseases society of America and the society for healthcare epidemiology of America guidelines for developing an institutional program to enhance antimicrobial stewardship. Infect Dis Clin Pract.

[35] Smith MJ, Gerber JS, Hersh AL (2015). Inpatient antimicrobial stewardship in pediatrics: a systematic review. J Pediatric Infect Dis Soc.

[36] Pan-Canadian Public Health Network. The Communicable and Infectious Disease Steering Committee Task Group on Antimicrobial Use Stewardship. *Antimicrobial Stewardship: Final Report to the Public Health Network Council*. http://phn-rsp.ca/pubs/anstew-gestan/pdf/pub-eng.pdf (2016).

[37] Wong J (2018). Canadian pediatric antimicrobial stewardship programs: Current resources and implementation characteristics. Infect Control Hosp Epidemiol.

[38] World Health Organization. Antimicrobial resistance surveillance. https://www.who.int/medicines/areas/rational_use/AMR_Surveillance/en/ (2019).

[39] Dalton BR (2015). Antimicrobial use over a four-year period using days of therapy measurement at a Canadian pediatric acute care hospital. Can J Infect Dis Med Microbiol.

[40] Bonnett J, Dalton B, Rajapakse N, Dersch-Mills D. Surveillance of Antimicrobial Utilization at Five Neonatal Intensive Care Units in an Urban Centre in Alberta, Canada. In Open Forum Infectious Diseases vol. 2 (Oxford University Press (OUP), 2015).

[41] Canadian Institute for Health Information. *Inpatient Hospitalizations: Volumes, Length of Stay and Standardized Rates*. apps.cihi.ca/mstrapp/asp/Main.aspx (2020).

[42] WHO Collaborating Centre for Drug Statistics Methodology. ATC/DDD Index. *ATC/DDD Index* https://www.whocc.no/atc_ddd_index/.

[43] Pakyz AL, Gurgle HE, Ibrahim OM, Oinonen MJ, Polk RE (2009). Trends in antibacterial use in hospitalized pediatric patients in united states academic health centers. Infect Control Hosp Epidemiol.

[44] D’Amore C (2021). Use of multiple metrics to assess antibiotic use in Italian children’s hospitals. Sci Rep.

[45] Brogan TV (2018). Variability in Antibiotic Use Across PICUs. Pediatr Crit Care Med.

[46] Gerber JS (2010). Variability in antibiotic use at children’s hospitals. Pediatrics.

[47] Blinova E (2013). Point prevalence survey of antimicrobial utilization in the cardiac and pediatric critical care unit. Pediatr Crit Care Med.

[48] Fischer JE, Ramser M, Fanconi S (2000). Use of antibiotics in pediatric intensive case and potential savings. Neonatal Pediatr intensive care.

[49] Renk H (2020). Antibiotic stewardship in the PICU: impact of ward rounds led by paediatric infectious diseases specialists on antibiotic consumption. Sci Rep.

[50] Le Saux N (2014). Canadian Paediatric Society - Infectious Diseases and Immunization & Committee: Guidelines for the management of suspected and confirmed bacterial meningitis in Canadian children older than one month of age. Paediatr Child Heal.

[51] BC Children’s Hospital & PHSA Antimicrobial Stewardship Program. *PEDIATRIC EMPIRIC ANTIMICROBIAL GUIDE 2016*. https://www.childhealthbc.ca/sites/default/files/PediatricEmpiricAntimicrobialGuide2016.pdf (2016).

[52] Koopmans LR, Finlayson H, Whitelaw A, Decloedt EH, Dramowski A (2018). Paediatric antimicrobial use at a South African hospital. Int J Infect Dis.

[53] Audry-Degardin E, Dubos F, Leteurtre S, Beaucaire G, Leclerc F (2007). Évaluation de la prescription antibiotique dans un service de réanimation pédiatrique. Arch Pédiatrie.

[54] Balkhy HH (2019). Antimicrobial consumption in three pediatric and neonatal intensive care units in Saudi Arabia: 33-month surveillance study. Ann Clin Microbiol Antimicrob.

[55] Kazzaz YM (2020). Evaluating antimicrobial appropriateness in a tertiary care pediatric ICU in Saudi Arabia: a retrospective cohort study. Antimicrob Resist Infect Control.

[56] Araujo da Silva AR (2020). Patterns of antimicrobial consumption in neonatal and pediatric intensive care units in Germany and Brazil. Eur J Clin Microbiol Infect Dis.

[57] Di Pentima MC, Chan S (2010). Impact of antimicrobial stewardship program on vancomycin use in a pediatric teaching hospital. Pediatr Infect Dis J.

[58] Mahmoud A, Al Saif S, Baylon B, Balkhy H, Al Banyan E (2014). PO-0549 Antimicrobial Use In Neonatal Units At King Abulaziz Medical City, Riyadh, Ksa, Prospective Observational Study. Arch Dis Child.

[59] Levy ER, Swami S, Dubois SG, Wendt R, Banerjee R (2012). Rates and Appropriateness of Antimicrobial Prescribing at an Academic Children’s Hospital, 2007–2010. Infect Control Hosp Epidemiol.

[60] Cantey JB, Wozniak PS, Pruszynski JE, Sánchez PJ (2016). Reducing unnecessary antibiotic use in the neonatal intensive care unit (SCOUT): a prospective interrupted time-series study. Lancet Infect Dis.

[61] Galankin TL (2018). Retrospective surveillance of antibiotic use in maternity wards and neonatal intensive care units in Saint Petersburg. Russia Eur J Clin Microbiol Infect Dis.

[62] Polk RE, Fox C, Mahoney A, Letcavage J, MacDougall C (2007). Measurement of adult antibacterial drug use in 130 US hospitals: comparison of defined daily dose and days of therapy. Clin Infect Dis.

[63] Dalton BR, Sabuda DM, Bresee LC, Conly JM (2015). Assessment of antimicrobial utilization metrics: days of therapy versus defined daily doses and pharmacy dispensing records versus nursing administration data. Infect Control Hosp Epidemiol.

